# Subjectivity and Non-Objectifying Awareness

**DOI:** 10.1007/s13164-022-00665-7

**Published:** 2022-12-09

**Authors:** Donnchadh O’Conaill

**Affiliations:** Département de Philosophie, Université de Fribourg, 20 Av. de L’Europe, 1700 Fribourg, Switzerland

## Abstract

We are each aware of our own experiences as they occur, but in this inner awareness our experiences do not seem to be presented to us as objects in the way that they typically are when we reflect on them. A number of philosophers, principally in the phenomenological tradition, have characterised this in terms of inner awareness being a non-objectifying mode of awareness. This claim has faced persistent objections that the notion of non-objectifying awareness is obscure or merely negatively characterised. In this paper I shall outline a positive conception of a non-objectifying mode of awareness, feature-encountering awareness. I shall apply this conception to our awareness of our experiences, characterising it as an awareness of instantiations of phenomenal properties in a temporal dimension. Inner awareness thus characterised clearly differs from standard modes of objectifying awareness.

It is widely thought that each subject is necessarily aware of her experiences as they occur, in a way which does not require reflecting upon or attending to them.^1^ In what follows I shall term this non-reflective awareness the subject’s *inner awareness*. Inner awareness is often said to constitute the subjectivity of our experiences, their distinctively first-personal character (Zahavi 2005, 122; Kriegel 2009¸ 8).

There are different conceptions of inner awareness, e.g., as perceptual in nature (Lycan 1996), as a kind of thinking (Rosenthal 1997), or as a sui generis form of self-representation (Kriegel 2009). While these accounts differ as regards the *mode* of inner awareness, there are important similarities between them as regards its *content*. Specifically, each of these accounts treats conscious experiences as the intentional objects of inner awareness, and as appearing to the subject in much the same way as other intentional objects. However, a rival approach rooted in the phenomenological tradition rejects this assumption. Proponents of this approach accept that subjects have a characteristic awareness of their own experiences but deny that this awareness is best understood along the lines of our awareness of objects: “when we are absorbed or immersed in our daily concerns and simply live through our experiences, they are not given as objects; they are not something we observe from a distance and they do not stand opposite us” (Zahavi 2005, 64).^2^

While this approach has been widely adopted in the phenomenological literature, it has faced persistent criticisms, in particular the objection that it is obscure and at best characterises inner awareness negatively. The burden of this paper is to address these objections: to provide a positive account of what it is to be aware of experiences but not be aware of them as objects.

More precisely, this paper has two aims. The first is to provide a positive account of non-objectifying awareness, i.e., a positive account of a mode of awareness which clearly fits certain criteria for being non-objectifying. The second aim is to use this account to clarify how inner awareness can be non-objectifying. This second aim is rather modest: it is to provide a constructive positive account of inner awareness which captures a feature of it which, though not uncontested, has been accepted by many philosophers. I shall not argue that this account of inner awareness is to be preferred overall, merely that it is coherent and has some plausibility.

In section 1, I sketch some phenomenological differences between inner awareness and reflective awareness of one’s experiences. In section 2, I outline three problems thought to face non-objectifying inner awareness. In section 3, I sketch a conception of objectifying awareness which allows us to extract criteria for non-objectifying awareness. In section 4, I outline the notion of a feature-encountering mode of awareness. In section 5, I consider how inner awareness can be understood as a feature-encountering mode of awareness. In section 6 I outline how inner awareness thus characterised fits the criteria of non-objectifying awareness stated in section 3. Finally, in sections 7 and 8 I consider two further issues: whether inner awareness on this conception is an awareness of token experiences, and whether it is compatible with an awareness of oneself as a subject of experiences.

## Inner Awareness and Reflection

Before considering an account of non-objectifying awareness, it is worth saying more about why one might think that inner awareness differs at least from many familiar cases in which one is aware of objects. The primary reason one might think this is based on a contrast between reflection and inner awareness. Various differences between reflection and inner awareness have been noted; for example, reflection is rare, usually voluntary, usually effortful, and typically involves focusing one’s attention on oneʼs own experiences, whereas inner awareness is ubiquitous, involuntary, effortless and is a marginal form of awareness (Kriegel 2009, 49–50). But in addition to these differences, it seems there is a difference between how experiences appear in reflection and how they typically appear as one undergoes or lives through them.

It is sometimes suggested that in reflection one ʻsteps backʼ from oneʼs experiences, viewing them ʻfrom a distanceʼ, as it were (Zahavi 2005, 64). This might seem to involve self-division or self-alienation (Gallagher & Zahavi 2008, 61, 64). These metaphors are suggestive but cannot bear too much weight. When I reflect, I am typically aware that it is my own experience of which I am aware; this is an important limit to the degree to which reflection typically involves anything like self-division or distancing oneself from one’s experiences.

Nevertheless, I think these metaphors indicate something important: in reflection, we are *confronted by* and can *observe* our experiences, in a way which we cannot do in inner awareness. In reflecting on an experience, one can think of it precisely as a particular event, as located and demarcated in various ways. For instance, in recollecting a past experience you can place it in time relative to other experiences and consider when it began and when it ended. More generally, in reflection one can pick out particular experiences, distinguish them from each other, and can explore different features of the same experience as one brings them to mind: e.g., how quickly did the feeling of embarrassment dawn? Did you feel embarrassed at the same time or just after you felt some other emotions? The point can be put like this: in reflection one can *cognitively grasp* particular experiences as distinct entities whose features one can explore. It is in this sense that one can *observe* oneʼs experiences in reflection.

This point should not be overstated. Many experiences do not have distinct, objective boundaries in the same way that ordinary objects of perception like trees or cars do (Cassam 1997, 109). A sense of frustration or a feeling of unease can creep up on one in such a way that it is very difficult even when reflecting to say when it began or ended. And to some degree at least the way experiences are individuated and distinguished is a matter of our specific concepts and interests, e.g., how we categorise them, the questions we ask of them, and so on.

That said, there are often relatively clear differences between distinct experiences, for instance differences in their objects (seeing a dog as opposed to seeing an airplane) and their modes (hearing the dog as opposed to seeing it or remembering having seen it). Many experiences do have distinct boundaries, and can be given as bounded and distinct in reflection. And in reflecting on any sequence of experiences, we can consider how we might draw distinctions within it and around. To draw such distinctions between experiences is to cognitively grasp them, even if this grasp is to some extent arbitrary.

In contrast, as one lives through experiences one does *not* grasp them in this way. At any rate, they are not typically given as demarcated or bounded. Furthermore, in undergoing an experience one is not thereby aware of it as a single entity with different features which one can explore in different ways. In inner awareness, one does not confront oneʼs own experiences as observable entities.

Dan Zahavi describes the way experiences are given to us in similar terms. When we live through experiences,

consciousness does not appear to itself chopped up in bits. It is nothing jointed; it simply flows […] The relation between two acts must rather be likened to the relation between two waves in the same stream than to two wagons in the same train. […] It is only due to a special apprehension, namely, when we theamatize the acts, that they are constituted as enduring objects in subjective time. When we reflect, we impose a new temporal form upon our experiences, they are made into subjective objects and posited in or injected into sequential time (1999, 77; see also 2005, 66).

This ʻnew temporal formʼ is precisely that of demarcated and bounded particulars:

When my intentional act is given as an identity across differences, when it is given as a clearly demarcated enduring unity in a manifold of temporal phases, and as something with a temporal location that one can return to again and again, it is given as a temporal object (1999, 76; see also 2011, 17-18).

The metaphor of waves in the stream, as opposed to wagons in a train, suggests that in inner awareness my experiences are not given as a series of discrete elements or units, neatly bounded and distinguished from each other.^3^ That said, the flow of experiences is given as differentiated, rather than as “a homogeneous gruel in which nothing stands out” (Brough 2011, 33). The suggestion is that I am aware of variation in my experiences as I live through them, but they are not given to me as discrete particular entities.

## Objections to Non-Objectifying Awareness

Phenomenologists have characterised what I have termed inner awareness in a number of ways. A key idea running through these different descriptions is the notion of *pre-reflective self-awareness*, “an immediate, implicit, irrelational, nonobjectifying, non-conceptual and non-propositional self-acquaintance” (Zahavi 1999, 33).^4^ In contrast, reflective self-awareness is characterized as “an explicit, relational, mediated, conceptual, and objectifying thematization of consciousness” (*op. cit.*).

The notions of pre-reflective self-awareness and more specifically of non-objectifying awareness have been subjected to three main lines of criticism. The first is that these notions are *obscure*. For instance, Christopher Hoerl says of the notion of non-objectifying awareness, “On the face of it, this notion seems to be in at least as much need of further elucidation as the idea of pre-reflective self-awareness. Thus, it is not clear how much further illumination we gain from trying to make sense of the latter in terms of the former” (2013, 388; see also Kriegel 2009, 103 and Musholt 2015, 6, for similar points about pre-reflective self-awareness).

Second, the characterisations of pre-reflective self-awareness and of non-objectfying awareness which have been offered are generally negative. Consider Zahavi’s gloss on pre-reflective self-awareness as “immediate, implicit, irrelational, nonobjectifying, non-conceptual and non-propositional” (1999, 33). Every one of these characterisations (with the possible exception of ‘implicit’) is negative. The worry is that we have not been given a *positive account* of this kind of awareness, stating what it is as opposed to what it is not. Kristina Musholt suggests that “we are left with a purely negative characterization of the phenomenon […] we have yet to learn how to make sense of self-consciousness in terms of a positive characterization” (2015, 6). Likewise, Jonathan Schear writes that “when it comes time to offer a positive description of this form of consciousness, we are told, for example, that it is a ‘subtle background presence’” (2009, 103, quoting Zahavi 2005, 124; see also Kriegel 2009, 101).^5^

Third, the notion of non-objectifying self-awareness has been thought to be self-contradictory. Uriah Kriegel points out that inner awareness is supposed to be an awareness *of* something, i.e., each subject’s experiences. He continues “it strikes me as conceptually true that, in the relevant sense of ʻobjectʼ, awareness-of is always awareness-of-object”, concluding “I cannot really wrap my mind around the notion of non-objectifying awareness” (2009, 105–106).

To some extent the third objection can be considered independently of the other two. The dispute here concerns (a) what counts as an *object* of awareness, and (b) what counts as an *objectifying* mode of awareness. Kriegel understands (a) in very broad sense: the relevant sense of object is “that-which-an-intentional-act-is-about” (*ibid.*, 105 n. 8), or that of which one is aware. When ʻobjectʼ is understood this way, Kriegel`s conceptual claim (awareness-of is always awareness-of-object) seems correct. However, Zahavi has a narrower notion of ʻobjectʼ in mind: “From a phenomenological perspective, however, objecthood is a specific mode of givenness. For Husserl, an object is something that is constituted in a process of objectification” (2005, 64).^6^ That is, Zahavi is restricting (a) by appeal to (b). Implicit in this restricted notion of ʻobjectʼ is a contrast with entities which are *not* given in this way, that is, which are not constituted in a process of objectification, but of which one is nevertheless aware. Since Zahavi and Kriegel are operating with different notions of ʻobjectʼ, Kriegel’s conceptual claim and Zahavi’s notion of non-objectifying awareness are not mutually exclusive.

In offering this response to the third objection, I have not yet clarified what it is for something to be given in either an objectifying or a non-objectifying way. Until some account of this difference is provided, the response I have just offered is provisional. To clarify these matters would in effect be to respond to the first two objections. The remainder of this paper in effect offers just such a response.

## Objectifying Awareness

To understand non-objectifying awareness, we should start by trying to get a better handle on objectifying awareness, on what it is for something to be given as an object. Zahavi, following Husserl, describes it as follows:

something is given as an object only the moment it is experienced as being in possession of a sort of *transcendence*. It is only when we experience something as a unity within a multiplicity of adumbrations, or as an identity across differences, that is, as something that transcends its actual appearance or that can be intended as the same throughout a variety of experiential states, that we experience it as an object (2005, 64).

Husserl himself glosses an objectified entity as

that which is identified in distinct acts which form a synthesis; in this synthesis we are aware of it as the same, as that which can always be recognized, or also as that which is freely repeatable in recollections or freely producible in perceptions (when we go there and take one more look) (1973, 62).

These descriptions apply most straightforwardly to perceptual experiences of concrete material entities, e.g., tables, trees or dice. When one sees a table, it is typically given as a single discrete entity with a number of different features (a specific colour, shape, etc.), and as remaining the same through successive experiences (i.e., as one examines it from one angle and then another, or when one first sees it and then touches it). That is, the entity is implicitly *identifiable* as the numerically identical bearer of various properties, and *re-identifiable* as the numerically identical object of successive experiences.

In addition, in perceiving the table one will have a sense of this entity as having *further features*, features which are not themselves given in any specific experience (for instance, sides which are not visible from this angle). The sense of transcendence also involves a sense of these further features as open to being presented in further experiences; for instance, the sides of the table which are not currently visible to me would become visible if I was to look at it from a different angle. And again implicit in this sense of the further features being available to further experience is a sense of the entity itself as *re-identifiable*; the very entity which I now see is the one which could be further revealed in further experiences, and in principle I could be aware of it as the same throughout these further experiences.

Things are slightly more complicated when we consider entities belonging to other ontological categories, such as events, states or processes. Events are not always given in perception as discrete entities, that is, as bounded and distinguishable from each other. For instance, while events typically appear in perception as spatially located, their spatial boundaries are not always distinct (or not as distinct as the boundaries of material entities typically are). Consider perceiving someone placing a glass on a table. To some extent it is an arbitrary matter whether this event includes the person’s moving their upper arm, bending their torso or visually following the movement of the glass. At any rate, it is not as though when one sees this event, certain movements are given as belonging to it and certain movements are not, in the way that, e.g., the distinction between the glass and the table is clearly visible.

Nevertheless, many of the points mentioned about perceiving the table apply here also. Events are typically given as single entities with different features (that is, they can be qualified in various ways, e.g., the glass might be placed slowly or quickly, heavily or lightly, etc.).^7^ When one picks out an event, for instance by visually attending to it, one is often in a position to examine it more closely, to consider different facets, to return to it in further experiences (recollecting having seen it, asking theoretical questions about it, etc.). And this process can also involve distinguishing the event more carefully from others, i.e., drawing its spatial and temporal boundaries (or at least posing the question of where its boundaries lie). These characteristics of event perception are very similar to how I have suggested experiences are given in reflection.

The details of objectifying awareness are worked out by Husserl in a number of analyses, most famously of perceptual experiences (e.g., 1982). For present purposes, an objectifying mode of awareness can be characterized as a mode of awareness each instance of which satisfies each of the following conditions:

(i) the object is given as a single entity to which various features belong;
(ii) the object is given as a single entity in different phases of experience;
(iii) the object is given as having further features beyond those which are directly given at this moment (in this experience or phase of experiencing);
(iv) the object is given as having further features which can in principle be directly given in further experiences of this same object.

This conception of objectifying awareness helps to develop my response in the previous section to the third objection. Specifically, from the criteria for objectifying awareness stated above we can extract criteria for non-objectifying awareness, i.e., experiences which lack one or more of (i)-(iv) (for a similar approach see Textor 2017, 177–180). What is now needed is to clarify what an experience which lacks this sense of transcendence – one which does not satisfy some or all of conditions (i)-(iv) – would be like.

## Feature-Encountering Awareness

In order to develop a positive account of a non-objectifying mode of awareness, I shall outline a conception of a mode of awareness in which one is aware of general features being instantiated in specific locations. I shall term this a *feature-encountering mode of awareness*.^8^ In such a mode of awareness, *what* one is aware of may well be re-identifiable particulars, but one will not be aware of them *as* re-identifiable.^9^ Rather, they will appear to one as instances of general features located within a certain dimension.

Simple instances of a feature-encountering mode of awareness involve two elements: first, an awareness of the feature one is encountering, and second, an awareness of its location in the relevant dimension. I take ‘feature’ to include any general type of entity or any respect in which entities can be similar or distinct. Features can be properties, but also kinds of stuff (e.g., gold or snow). The dimension is the range of possible locations within which one can encounter the relevant feature. For example, in Austen Clark’s model of sensory awareness, the dimension within which features are located is the array of ʻplace-timesʼ, space–time regions of finite extension (2000, 81). Instances of this kind of awareness will have contents of the form ‘Red here’, ‘Chirping there’, etc. The terms ‘here’ and ‘there’ will indicate the location of the feature at certain space–time regions.

The conception of a feature-encountering mode of awareness involves both negative and positive claims. On the negative side, one cannot in this mode of awareness *re-identify* the same instance of the same feature at different locations, as opposed to being aware of the same feature at different locations. That is, one cannot in this mode of awareness distinguish between the same particular encountered at different locations, and different particulars of the same type (i.e., different instances of the same feature) encountered at different locations. So, for example, in a feature-encountering mode of awareness one cannot track the same particular from one location to another. It is feasible that in this mode of awareness one can register that the scene one is aware of is varied at any one time, and one can register the scene changing across time (i.e., that the features one encounters at certain locations differ from those encountered at those locations previously).^10^ But one cannot be aware of this change as involving the same particular being first at one location and then another.^11^

We can also offer a positive characterization of this mode of awareness. One who undergoes instances of it will be aware of general features, aware of their location in a certain dimension, and (as indicated in the paragraph just above) aware of differences between features at different locations and of differences across time in which features are at which locations. Depending on the range of different features to which one is sensitive, one can thereby be aware of a considerable amount of variety and sameness.

## Inner Awareness as a Feature-Encountering Mode of Awareness

I suggest that inner awareness can be understood as a feature-encountering awareness of the subjectʼs own experiences. On this view, the features one encounters in inner awareness are *phenomenal properties*. Phenomenal properties are properties the instantiation of which help to constitute what it is like for subjects to have experiences (Nida-Rümelin 2018, 3362–3363). There are two main conceptions of the ontology of token experiences. On the first view, token experiences are identical with instantiations of phenomenal properties by conscious subjects. This is the *framework of experiential properties* (Nida-Rümelin *op. cit.*). On the second view, phenomenal properties are instantiated by experiences, understood as particulars which are not themselves subjects but which are suitably related to subjects (this is what Nida-Rümelin terms the *experience property framework*; for criticism of this framework see *ibid.*; Taylor 2020). In what follows I shall assume the framework of experiential properties, but later in this section the choice between these two frameworks will be relevant and I shall discuss it briefly.

Exactly which phenomenal properties there are is a matter of some debate. On restrictive views, phenomenal properties are limited to sensory qualities (e.g., reddish qualities, qualities of feeling cold or hot, feeling painful or pleasurable, etc.). On more liberal views, phenomenal properties include properties distinctive of agentive experiences, conscious cognition, and complex perceptual properties such as the visual appearance of a guitar or a jackdaw.^12^ I shall adopt a liberal view, but the discussion which follows can if necessary be reformulated in more conservative terms.

Providing a positive account of the dimension or dimensions within which one encounters phenomenal properties is more difficult. The first thing to note is that they must be encountered in a *temporal* dimension. That is, insofar as I am aware of my experiences by having them, I am aware of phenomenal properties as present at certain times. My account of this dimension will be brief, since it has been described extensively in the phenomenological literature on inner time-consciousness. It is standardly thought that one’s awareness of one’s experiences is tensed (e.g., I may have an awareness ʻNow it is painfulʼ). This awareness is also standardly thought to have two distinct axes: an awareness of simultaneity (e.g., ʻNow it is painful and it seems redʼ), and an awareness of temporal succession (e.g., ʻIt is painful againʼ, ʻPain is increasingʼ). The awareness of succession requires that one be aware, not only of what one is experiencing at this very instant, but of what one has just experienced (and perhaps what one is about to experience). Inner awareness thus takes in an extended or specious present.^13^

The view that this is the only dimension in which phenomenal properties are encountered in inner awareness is plausible. On this view, inner awareness just is an awareness of phenomenal properties as instantiated at certain times. I shall term this the *one-dimensional view* of inner awareness. That said, some might offer reasons in favour of there being other dimensions. In the remainder of this section I shall criticize three arguments supporting what I shall term the *two-dimensional view*, the view that there is a single extra dimension which is spatial in nature.^14^

First, it might be thought that a spatial dimension is needed to deal with perceptual experiences. Consider an experience of seeing a blue car. This experience has a phenomenal character which, I assume, involves the following: it is a visual appearing of an object with certain color properties, which appears as located at a certain distance and orientation (relative to a *point of view*, a spatio-temporal position which is not itself visually presented but which helps to structure how things appear in the experience – see, e.g., Horgan and Nichols, 2016, 148). That is, the phenomenal character of this experience is spatial. The scene I see is presented as being spatial, and in a sense the presentation itself has a spatial character (e.g., it is structured spatially around a point of view).^15^ So, the thought might go, in being aware of a visual experience with this specifically spatial phenomenal character, surely one must be aware of it as in a spatial dimension?

To address this worry, we need to distinguish two instances of awareness which this experience involves: (1) my awareness of the objects of the experience, i.e., the entities which I see (and perhaps their visible properties), and (2) my awareness of this experience in inner awareness, specifically my awareness of the phenomenal character of this experience, of what this experience is like.^16^ It is (2) which I am suggesting can be understood as a feature-encountering mode of awareness. So the issue we are presently concerned with is whether or not my awareness of the phenomenal character of this experience is itself spatial in nature, in the sense that it involves encountering phenomenal properties in a spatial dimension.

What it is like for me to have this experience involves a specific *phenomenal content*: that is, certain entities are presented in the experience as being certain ways (see, e.g., Schellenberg 2011, 723; Chudnoff 2013, 565–566). These ways include spatial features (e.g., the apparent size and location of the car). What it is like for me to have this experience also involves a certain *phenomenal mode*, in this case vision. The visual mode of awareness is itself spatial: in a visual experience, entities are presented relative to a point of view. The experience has these spatial characteristics in virtue of the phenomenal properties whose instantiation constitutes the phenomenal character of the experience. That is, they are properties which, when instantiated, present entities as spatial, and do so in a spatially structured way (e.g., appearing as located relative to a point of view).^17^ But note that these characteristics qualify (1) rather than (2); they are a matter of my awareness of the visual scene rather than my awareness of my experience of this scene. And they seem to exhaust the spatial character of the experience as described in the previous paragraph. Therefore, it is not clear why my awareness of the experience, i.e., (2), would require further spatiality.

Furthermore, these phenomenal properties do not themselves seem to be given as spatially located. They certainly are not visually presented in the scene I see. One might think that they might nonetheless be spatially presented, at the point of view relative to which what I see is located. But this does not seem correct either. The fact that the experience presents its objects in a spatially structured way does not require that the phenomenal properties must themselves appear as located within the spatial structure. The experience can be said to involve a point of view because it has what Horgan & Nichols term *zero point representational structure*. This kind of structure characterises non-phenomenal as well as phenomenal representations: “Picture-like analog representations, for instance, have built-in zero-point structure: they represent a scene from a point of view—a point of view that does not get represented itself” (*op. cit.*, 161).

A second argument for a spatial dimension appeals to a version of the *many-property problem* (Jackson 1977). Consider the following two visual experiences: one is of a red square beside a blue triangle, the other is of a blue square beside a red triangle. These experiences are phenomenally different, but they seem on the face of it to involve the same phenomenal properties (colour properties, shape properties, relative spatial location properties). Since these experiences are phenomenally different they would presumably be distinguishable in inner awareness, but how can this be explained if inner awareness is just an awareness of property-instantiation, and if each experience involves the same phenomenal properties?

The distinction drawn above between (1) and (2) also allows us to deal with this worry. The many-property problem does arise with respect to (1), our awareness of the objects of our experiences. It indicates the need for visual experiences to include elements beyond the phenomenal properties listed in the previous paragraph. So, for instance, each colour property might be presented as located at different spatial points in the different experiences, or as belonging to different property-bearers. That is, in addition to the phenomenal properties listed in the previous paragraph, each of these visual experiences will involve further phenomenal properties, e.g., the property of presenting a specific entity as red and triangular. The phenomenal difference between the two experiences will be exhausted by the differences between these extra properties. And in that case the problem will not arise with respect to (2). These experiences will be distinguishable in inner awareness because there are differences in which phenomenal properties each involves. There is no need for a further dimension within which the phenomenal properties that each experience involves will differ in location.

The third and strongest argument for the two-dimensional view appeals to certain *bodily experiences*, e.g., sensations such as feeling a pain or an itch. What is particularly relevant is that the phenomenal character of such experiences typically involves a *felt location*; they seem to occur at a specific bodily location (e.g., I feel pain in my knee).^18^ One way to understand the felt location of these experiences is that in having them one is aware of phenomenal properties (e.g., *feeling pain*, *feeling cold*) which are presented as themselves located at these parts of the body. This seems to be the account of felt location offered by, e.g., Tim Crane: “The qualities of which we are aware in bodily sensation – the sensory qualities of hurting, feeling cold or warm and so on – are predicated in these experiences of parts of the body” (1998, 237; see also Brewer 1995, 297–300; Martin 1995, 268–269). On this view, when I feel pain in my knee, a phenomenal property, *feeling painful*, is encountered as itself spatially located in my knee.

If this view is correct, it would require that for many phenomenal properties, to be aware of them in inner awareness requires being aware of them as bodily and so as spatially located. Furthermore, this might be thought to threaten the conception of inner awareness as feature-encountering. Proponents of this view of felt location have argued that it entails that in bodily awareness we are aware of our bodies as bounded entities located in a larger space (see Martin 1995, 270–273). One way to interpret this is that felt location requires awareness of the body as a re-identifiable particular, in a way which seems incompatible with any feature-encountering awareness (I shall discuss a similar worry in section 8).

I think that these bodily experiences can be accommodated by the one-dimensional view in much the same way as visual experiences. The relevant phenomenal properties determine the phenomenal content of bodily experiences. This content is itself spatial, and this is what accounts for felt location (more on this presently); but the subject’s awareness of these phenomenal properties being instantiated is not itself spatial. That is, in inner awareness of bodily experiences one is not aware of instances of phenomenal properties as themselves located in a bodily spatial dimension.

Before outlining some ways in which this account of bodily experiences can be developed, I shall address the phenomenological claim I want to reject, i.e., the claim that in undergoing bodily experiences one is thereby aware of phenomenal properties as themselves located at specific body-parts. This phenomenological claim suggests the following metaphysical conception of how phenomenal properties are instantiated in bodily experiences: “In bodily awareness, one is aware of determinately spatially located properties of the body that are also necessarily properties of the basic subject of that very awareness. In contrast with external sense perception, a psychological property of oneself is physically located in or on the body, as a property of the body” (Brewer 1995, 300).

Whether or not this metaphysical conception is coherent will depend on certain background assumptions, in particular the choice between the two ontological frameworks for token experiences distinguished at the start of this section. Suppose we assume Nida-Rümelin’s framework of experiential properties, according to which each token experience is identical with the instantiation by a subject of a phenomenal property. Now consider experiencing a pain in one’s knee. Given the framework of experiential properties, this token experience is identical with a subject’s instantiating a phenomenal property, *feeling painful*. But my knee is not itself a subject. There is nothing it is like for my knee to feel painful. Rather, for my knee to feel painful is for there to be something it is like for me. So it is not clear that the relevant phenomenal properties could be instantiated by that part of my body.^19^

Perhaps one might respond that the property *feeling painful* is instantiated by me *at* a certain part of me, my knee. We are familiar with this idea in other contexts. A poker can have the property *being hot* and, at the same time, *being cold*: it instantiates each property at a different part. But in this case, it is clear what it is for a part of a poker to instantiate the property *being hot* (or, at any rate, it is just as clear what this is as what it is for the poker itself to instantiate this property). The problem is that given the framework of experiential properties, it is not clear how my knee could itself instantiate the phenomenal property *feeling painful* (assuming that my knee is not itself a subject). Furthermore, one can experience pain in a body part which does not exist, e.g., in a phantom limb. Here the experience has a felt location, but we surely are not obliged to say that a non-existent limb itself instantiates the relevant phenomenal property.

Another possible response would be to say that the body parts *seem* to instantiate phenomenal properties, even though they do not. This would require a bodily dimension within which one encounters these properties, and so would count as a two-dimensional view. But this still leaves us with the question of what it would be for a body part to *seem* to instantiate a phenomenal property. And again, given the framework of experiential properties, it is no easy task to say what it would be for a body part to seem to instantiate such a property (particularly if one does not mistakenly take the body part to be a subject, as is surely the case when I feel pain in my knee).

Things do seem different if we assume the other ontological framework for token experiences, the experience property framework (on which each token experience is an event which is not identical with a subject and which itself instantiates phenomenal properties). This framework at least allows for the possibility of being aware of phenomenal properties as themselves located at specific body-parts. The suggestion would not be that the body-part itself (e.g., my knee) instantiates the phenomenal property (since my knee is presumably not identical with any token experience). Rather, the thought might be that the experience is identical with an event which occurs *in* my knee, and in being aware of this event as occurring in my knee I can become aware of the relevant phenomenal property, feeling painful, as located *at* my knee. So the experience property framework is at least compatible with the argument from bodily experiences in support of two-dimensionalism.

This point is important, but it should not be overstated. The experience property framework does not entail two-dimensionalism. Furthermore, if the argument for two-dimensionalism presupposes the experience property framework, a thorough defence of this argument would require in turn a proper examination of this framework, which would take us far beyond the scope of this paper. And there is the problem, noted above, of pains in phantom limbs. Such pains presumably have felt locations, but it is hard to see how we can account for their felt location in the way proposed in the previous paragraph, since we can hardly say that an event occurs in a non-existent body part.

We can avoid these difficulties by adopting an alternative approach to felt location, one which is compatible with the one-dimensional view. On this approach, the property *feeling painful* constitutes the phenomenal character of an experience which presents me with an object, my knee. In this experience, my knee is presented as being a certain way. But the phenomenal property is not itself presented as being located at the knee.

This approach can be developed in different ways. One way would be to hold that the phenomenal property qualifies the *mode* of my awareness. On this account, I am not aware of my knee as itself painful; it would be more accurate to say that I am painfully aware of my knee. Alternatively, the phenomenal property can be understood as the experience having a certain representational content. For instance, on an *evaluationist* theory of pain, the experience sensorily represents a disturbance in a certain body part, and represents this disturbance as bad (Bain 2013, 82). But this representational property is not itself represented as being located at the knee.

On either way of developing this approach, the phenomenal property *feeling pain* must be distinguished from what is localized, which is a different property: a *bodily sensible property*.^20^ Bodily sensible properties can be understood in different ways, e.g., the property *being damaged*, or the property *appearing to be damaged*.^21^ They could also be understood as similar to secondary qualities, i.e., essentially such that when a body part has one and is suitably related to a subject, the subject will have, e.g., a painful experience of that body part. So while bodily experiences might still be said to have felt locations, this would not involve locating phenomenal properties in any body parts. Therefore, one’s inner awareness of these phenomenal properties would not require a second, bodily dimension.

I am not claiming that this alternative approach to felt location is correct. To do so would require developing it in much more detail and contrasting it with rival views. What I am claiming is that there are prima facie coherent alternatives to the view of felt location which understands it as locating phenomenal properties at specific body parts. Since these alternatives do not seem to require a two-dimensional view, we are not forced to rule out the one-dimensional view because of felt location.

## Inner Awareness as Non-Objectifying Awareness

At the end of section 3, I summarised objectifying awareness as satisfying each of the following conditions:

(i) the object is given as a single entity to which various features belong;
(ii) the object is given as a single entity in different phases of experience;
(iii) the object is given as having further features beyond those which are directly given at this moment;
(iv) the object is given as having further features which can in principle be directly given in further experiences of this same object.

A non-objectifying mode of awareness would meet only some or none of these conditions.

What I termed a one-dimensional feature-encountering mode of awareness clearly does not meet condition (i). In this mode of awareness, one would be aware of features (e.g., phenomenal properties) instantiated at certain times. One would not be aware of one’s token experiences as single entities to which different features belong; at most, one would be aware of distinct phenomenal properties occurring simultaneously or successively.

Matters are not so straightforward regarding condition (ii). In this mode of awareness, one can be aware of the same phenomenal property being instantiated continuously over a period of time. A continuous instantiation of the same phenomenal property by a single subject just is a single temporally extended experience. So to be aware of a phenomenal property as continuously instantiated is arguably to be aware of an experience as a single entity through different phases of experiencing (that is, different phases of inner awareness).

However, even if this is granted, inner awareness satisfies (ii) only in this specific kind of case. One cannot have an awareness of a changing experience as a single entity; all one will be aware of is a succession of different phenomenal properties. As Mark Textor puts it, “I have first a ‘pressure’ headache, then I have a ‘pulsating’ headache. […] There is no awareness of one and the same headache that appears differently at different times” (2017, 180). So while inner awareness does seem to satisfy (ii), it does so in a way which is much more limited than in standard objectifying modes of awareness such as perception.

Something similar can be said of conditions (iii) and (iv). It may be that in inner awareness one would have a sense of further features. For instance, part of the temporal dimension of inner awareness may be what Husserl terms *protention*, a more or less indeterminate expectation of which features will be encountered next. To have a sense of such features as belonging to a specific token experience would require that one grasp this experience as a re-identifiable single entity. This will not be possible for many experiences, in particular any which involve changes in phenomenal properties (e.g., one’s experience of seeing a table from different angles, or hearing a car drive past). But in the specific case of an unchanging experience where one expects the same phenomenal property to continue to be instantiated, one might be said to have a sense of further features of the specific experience one is undergoing.

To summarise: inner awareness construed as a one-dimensional feature-encountering mode of awareness clearly does not satisfy condition (i), and at best satisfies conditions (ii)-(iv) only for specific kinds of experiences. It thus clearly differs from the standard objectifying modes of awareness described in section 3. This result vindicates the distinction drawn in section 1 between how experiences appear in reflection and how they appear in pre-reflective awareness. As noted in section 4, we can provide a positive characterisation of feature-encountering modes of awareness. Therefore, we have an answer to the second objection outlined in section 2; and granted the intelligibility of the notion of a feature-encountering mode of awareness, we have an answer to the first objection outlined there also.

##  Is Feature-Placing Inner Awareness an Awareness of Token Experiences?

In the next two sections I shall address two further issues which might be thought to arise for the conception of inner awareness as a feature-encountering mode of awareness. The first issue concerns whether this conception is still an account of inner awareness as characterised at the beginning of this paper. Inner awareness was said to be an awareness each subject has of each of her experiences as she has them. More specifically, it is an awareness of *token* experiences, particular events which a subject undergoes. The worry, simply put, is that a feature-encountering mode of awareness does not yield awareness of token experiences. Rather, it yields awareness of the properties instantiated in (or by) experiences. This idea is explored by Brie Gertler, who presents it as an alternative to the view that in inner awareness one is aware of one’s occurrent experiences (2012, 450).

While this would involve a revision of the initial notion of inner awareness, it might be that this is not too great a cost to bear. But there are ways to deal with this worry rather than giving up on inner awareness as initially characterised.

One way to respond to this worry begins by considering the difference between being aware of a phenomenal property which is instantiated and being aware of a specific instantiation of this property (i.e., a particular event, assuming once again the framework of experiential properties). One way to distinguish these forms of awareness is by holding that awareness of a particular event *e1* must meet certain epistemic critieria, for instance that one be able to distinguish *e1* from distinct events *e2*, *e3*, etc., or that one identify *e1* in such a way that one can re-identify it.

But there is reason to resist this conception of what it is to be aware of a particular event. It is not obviously correct even when applied to ordinary perception. As noted in sections 1 and 3, events do not always appear as clearly distinct from each other; drawing boundaries between them is often difficult and can be a somewhat arbitrary matter. Furthermore, it is at best debatable whether awareness of an entity which is in fact a re-identifiable particular requires being aware of it *as* re-identifiable (i.e., picking it out in such a way that one could in principle re-identify it). Consider riding a fast train rushing through a crowded underground station. You see the people thronging the platform, and they do not appear as an indistinguishable mass; but it is hardly true that you perceptually distinguish each person you see, such that you could re-identify each of them.^22^ Or consider hearing someone talking just too low for you to make out what they are saying. What you hear are the words she utters, but you will be unable to pick them out or say what they were, even if they were spoken in a language you understand.

Another way to resist this objection is to consider what is revealed to one in a feature-encountering mode of awareness. In such a mode of awareness one would be aware of features, but also of their patterns of variation and sameness at specific locations. One could report instances of such a mode of awareness not only by simply uttering terms for features (‘Red!’, ‘Pain!’), but by uttering a feature-placing statement expressing one’s awareness of the features as located (‘Red here’, ‘Pain increasing’).^23^ This suggests that one is aware precisely of the features as being at certain locations (and not at others). And since features being at these locations just is their being instantiated at these locations, this seems to be an awareness of instantiations of these features, i.e., of particular events. It is true that in a sense one is not thereby aware of these particular instantiations *as* particulars; one has not picked them out in such a way as to be able to re-identify them. Nevertheless, it is plausible that *what* one is aware of are these particulars.

## Inner Awareness and Awareness of the Subject

The notions of inner awareness and pre-reflective self-awareness are often linked with awareness of oneself as a subject of experiences. One way to explore this link is by asking whether, given that one is aware of one’s experiences as one has them, one is given in inner awareness as their subject.^24^ Depending on how this question is answered, it might be thought to threaten the account of inner awareness as feature-encountering. While I shall not be able to address this topic fully, I shall outline an answer to this question and sketch how I think my account of inner awareness can accommodate this answer.

There are experiences in which plausibly one is given as a subject in inner awareness, and experiences in which plausibly one is not. As an example of the former, consider feeling guilty. An experience of guilt is not reflective, in that it does not require reflecting on or attending to one’s own experiences (though often one will reflect on oneself or one’s own behaviour). But it involves an awareness of oneself as a subject: to feel guilty is to be aware of oneself as having done something wrong, and to be aware of oneself as feeling this way. So to have inner awareness of the phenomenal property, *feeling guilty*, as instantiated seems to require being aware of oneself as instantiating it.

I also think there are experiences where one does not seem to be given as a subject in inner awareness. There are few uncontroversial examples, but depending on one’s background views some or all of the following are plausible candidates: consciously thinking that history unfolds in cycles, wondering whether quarks might have parts, imagining the universe before life evolved, or desiring world peace. In none of these experiences does it seem obvious that one must be manifest as the subject who is thinking, wondering, etc. (for further discussion see O’Conaill 2019).

My preferred view, therefore, is that inner awareness does not in and of itself require awareness of subjects as such, but it allows for cases in which the subject is manifest. But it might seem that this view is not compatible with the conception of inner-awareness as a feature-encountering mode of awareness. In a pure feature-encountering mode of awareness there can be no awareness of any particular as re-identifiable (waiving the point about condition (ii) made in section 6).

While this is correct, it seems that in the specific case of inner awareness it might be possible to make a principled exception. After all, inner awareness is an awareness of occurrent experiences, and I assume that the experiences each subject is aware of in this mode will all belong to that very subject. Therefore, the entire universe of entities of which one can be aware in inner awareness is already arranged relative to a single particular, oneself. So there is some reason to think that inner awareness can accommodate a single *privileged particular*, the subject of these experiences. It would not follow that anything else could be given in this mode as a re-identifiable particular. Rather, in many instances of inner awareness one will be aware of oneself as having certain phenomenal properties (e.g., *feeling guilty*), but these experiences would not thereby be given as themselves re-identifiable. (To be more precise, the claims made in section 6 about conditions (i)-(iv) would apply to them in just the same way that they apply to instances of inner awareness in which the subject is not manifest.)

A different worry is that the response sketched in the paragraph above is not compatible with the one-dimensional view. The worry is that on this response, phenomenal properties would be encountered both in time and as instantiated by oneself. Instead of reporting instances of inner awareness by saying, e.g., ‘Red now’ or ‘Pain continues’, one could say ‘I see red now’ or ‘I continue to feel pain’. One would in effect become a second dimension of inner awareness.

But this worry is ill-founded. In the context of feature-encountering modes of awareness, a dimension is a range of *distinct* locations at which features can be encountered. On the proposal under consideration there is only one subject which can be manifest in any instance of that subject’s inner awareness. To be aware of phenomenal properties as instantiated by a specific subject is not to be aware of them as being at one location rather than any other. Even if one was to report instances of inner awareness using ‘I’, every use of this term would in effect designate the same location. The possibility that a given phenomenal property could be instantiated by some other subject cannot arise within inner awareness.

## Conclusion

I have outlined a positive conception of a non-objectifying mode of awareness, feature-encountering awareness, and applied this conception to our inner awareness of our experiences. Inner awareness thus characterised clearly differs from standard modes of objectifying awareness. I have defended this account of inner awareness against the suggestion that it is not an awareness of our token experiences, and I have argued that it is compatible with our being aware of ourselves as subjects in inner awareness.^25^

## Footnotes

1 While this view is widely held it is not unanimously accepted. For criticisms see Schear 2009; for a defence of this view see Zahavi & Kriegel 2015. By ‘experience’ I mean any mental state, event or process which is necessarily such that it is like something for a subject to be in it or undergo it.

2 Similar claims can be found throughout the phenomenological tradition, from Husserl (2001, 273) to Sartre (2004, 5). As shall become clear in section 2, there is a sense in which these claims are compatible with the thought that episodes of inner awareness have intentional objects, in the sense that they involve awareness *of* something, specifically experiences. The claim that inner awareness is non-objectifying is better understood as a claim about *how* experiences appear to be when one is aware of them in this way.

3 This may not be how Husserl understood the metaphor, and it is probably not how John Brough understands it (2011, 34). But it seems very close to Zahavi’s reading, both in his 1999 and more recently (2011).

4 This form of self-awareness is an awareness of one’s experiences (it is a form of *self*-awareness in that each experience is claimed to include an awareness of itself). Whether one is also thereby aware of *oneself*, the subject of one’s experiences, is a further question (see section 8).

5 Other characterisations of inner awareness as non-intentional are also limited in this respect, e.g., Nida-Rümelin 2017, 63; Hellie 2007 (see Gennaro 2016 for criticisms of Hellie on this score). One way to respond to the second objection would be to suggest that the notions of pre-reflective self-awareness and non-objectifying awareness be taken as primitive and not amenable to an informative positive characterisation. The thought might be that in order to grasp these notions, all that is needed are some relatively clear examples and some ways of contrasting these modes of awareness with other modes, e.g., reflective awareness. I think this line of thought is correct, but it does not take us very far. In particular, doubts would remain concerning theoretical adequacy; it is not clear whether a primitive notion characterised in largely negative terms can be used to account for other phenomena in a perspicuous way. At the very least, a notion characterised in this way is severely limited, and we would have strong reason to prefer an account which provides a positive characterisation.

6 This reading of Husserl has been contested; see, e.g., Brough 2011, 32. However, note that Brough himself distinguishes between “objectivating” and “nonobjectivating” instances of intentionality. Given this, it seems to me that Brough agrees with Zahavi that (contra what Kriegel suggests) the distinction between objectifying and non-objectifying awareness makes sense. The difference between Brough and Zahavi on this point (though not others) seems to me to be largely a matter of terminology.

7 This is not to suggest that the ontological structure of an event’s having different features is the same as that of a material entity’s having features. Nor is it to say that the way an event’s features appear will be the same in each respect as the way a material entity’s features appear (for instance, there will be important differences as regards perceptual constancy).

8 As the name suggests, this idea draws on Peter Strawson’s work on *feature-placing statements* (1959, 202–225; see also Clark 2000 for an account of primitive sensory awareness based on Strawson’s work). ‘Encountering’ is intended to register the passivity of this mode of awareness, as opposed to the activity of placing features by uttering statements.

9 This is not to suggest that they will appear to one as *not* re-identifiable. Rather, in this mode of awareness the question of whether or not they are re-identifiable will not arise. In this sense, a feature-encountering mode of awareness is primitive compared to our standard modes of perception and cognition.

10 Clark denies that in a feature-encountering mode of awareness one could be aware of variation over time, since he holds that such a mode of awareness lacks tense (2000, 83; 2004, 465). But it is not clear why we should accept this. There appears to be nothing in the conception of a feature-encountering mode of awareness which rules out an awareness of variation across time as well as at a time. Furthermore, insofar as feature-encountering modes of awareness are modelled on feature-placing discourse, there is some limited support in favour of my interpretation. Strawson gives tensed examples of feature-placing statements (‘Now it is raining’ – 1959, 202); he also gives an example of a nontensed feature-placing statement which expresses temporal awareness (‘cat again’ – *op. cit.*, 207).

11 There is an important caveat to this claim, which I shall consider in section 6 when discussing criterion (ii) of objectifying awareness.

12 For discussion of these different views see Bayne & Montague 2011; for defences of the more liberal view see Strawson 1994, Zahavi 2005, 116–119.

13 For further discussion of inner time-consciousness see, e.g., Husserl 1991; Zahavi 1999, 2011; Brough 2011. On the two axes see Husserl 1973, 73.

14 A further possibility which I shall not be able to discuss is that phenomenal properties can be encountered in the foreground or the background of a dimension of conscious attention, e.g., as closer or further away from the centre of one’s conscious field (on the link between attention and location in the conscious field see Watzl 2017; thanks to Julien Bugnon for suggesting this possibility).

15 To see how this mode of presentation is itself spatial, contrast this experience with my thinking, e.g., ‘There is a blue car ten feet away from Maude’. This thought represents a scene as spatially arranged, but the way the scene is represented is not itself spatially structured.

16 On this distinction between the two instances of awareness, see O’Conaill 2019, 332–334. The discussion in this section develops in more detail some claims made in this earlier paper.

17 There may be another way in which this mode of awareness is spatial. Christopher Peacocke posits *visual field properties*, properties such as being an experience in which the area of the visual field in which an object is presented has a specific size and shape (2008, 7). Visual field properties are not analyzable in terms of the representational content of an experience, but it is plausible that they belong to the phenomenal mode, i.e., they pertain to a token experience’s being a *visual* experience. (Thanks to an anonymous referee for suggesting that I discuss these properties.).

18 The bodily experiences I have in mind are what David Armstrong terms “intransitive bodily sensations”, experiences where no distinction seems possible between the experience and what it is an experience of (1968, 309–310). Other bodily experiences, such as proprioceptive experiences, do not seem to have a felt location (thanks to an anonymous referee for suggesting I distinguish between these different types of bodily experiences).

19 It is true that some theorists identify the experiencing subject with the whole body, or with a specific body part, e.g., the brain. But on the view under discussion, phenomenal properties can be attributed to all manner of body parts. Furthermore, even if the subject is identical with the body or the brain, it does not follow that in a bodily experience one is aware of phenomenal properties as themselves spatially located (even if in fact they are).

20 The notion of bodily sensible properties is similar in some respects to Alex Byrne’s notion of *sensible properties*, perceptible properties of non-mental entities, e.g., colours or shapes (Byrne 2011). Byrne notes that sensible properties are often confused with *sensory qualities*, properties of experiences. In effect, this is what I am suggesting may be happening when bodily sensations are described in terms of body parts themselves instantiating phenomenal properties. (Byrne is skeptical that there are any sensory qualities, and would presumably be skeptical of phenomenal properties as well.).

21 The account I am suggesting is similar to Armstrong’s account of intransitive bodily sensations (see fn. 18 above) in that it treats the location of such experiences as intentional, e.g., its seeming to me that a certain sort of disturbance is occurring in a certain part of my body (see Armstrong 1968, 314–316).

22 A referee suggested that you would see the people on the platform without seeing each person. But this is not necessarily true of the scenario I have in mind. I think in this scenario one could see at least many of the persons at the front of the platform in Fred Dretske’s sense of “non-epistemic seeing” (1969, 20), i.e., visually distinguishing each of them from their environment. This seems compatible with one not thereby being in a position to re-identify each of these persons.

23 See also the discussion of *Lichtenbergian reports* in O’Conaill 2019, 331.

24 This question is closely related to another, whether in inner awareness one’s experiences are given as one’s own, i.e., whether they are characterised by a sense of mineness (see, e.g., Zahavi 2005, 124). For scepticism that inner awareness necessarily involves a sense of mineness, see Guillot 2017; O’Conaill 2019. Furthermore, I do not assume that if an experience is given as one’s own, one must also be given as the subject of that experience. In principle at least, one might be aware that an experience is one’s own without being given oneself as its subject (Howell 2010, 479; thanks to Andrea Giananti for discussion of this point).

25 Thanks to Alessandro Salice, Elisa Bezençon, Julien Bugnon, Martine Nida-Rümelin and to two anonymous referees for their comments on earlier drafts, and to Clare MacCumhaill for several helpful suggestions. Thanks also to audiences at the Eidos postgraduate seminar (Durham University), the SIFA conference (University of Eastern Piedmont, Novara), the International Symposium for Swiss Philosophy (Basel), the European Society for Philosophy & Psychology conference (Rijeka), and the Institut Jean Nicod (Paris) for their insghtful comments.
